# DEC2 Blocks the Effect of the ARNTL2/NPAS2 Dimer on the Expression of PER3 and DBP

**DOI:** 10.5334/jcr.149

**Published:** 2017-08-11

**Authors:** Juri Olkkonen, Vesa-Petteri Kouri, Elina Kuusela, Mari Ainola, Dan Nordström, Kari K. Eklund, Jami Mandelin

**Affiliations:** 1 Department of Medicine, Clinicum, University of Helsinki and Helsinki University Hospital, Helsinki, FI; 2 ORTON Orthopaedic Hospital, Helsinki, FI; 3 Center of Inflammation, Department of Rheumatology, University of Helsinki and Helsinki University Hospital, Helsinki, FI

**Keywords:** Circadian, Inflammation, TNF, Rheumatoid arthritis, ARNTL2, NPAS2, DEC2

## Abstract

The expression of clock genes *ARNTL2, NPAS2* and *DEC2* are disturbed in rheumatoid arthritis, an autoimmune disease with circadian variation of symptoms. We have shown that TNF is a potent inducer of these genes. We investigated the regulation of *ARNTL2* and *NPAS2* by TNF and elucidated their effect on other clock gene expressions. Additionally, we studied the effect of *DEC1* and *DEC2* on *ARNTL, ARNTL2* and *NPAS2*. Cultured primary human fibroblasts were stimulated with TNF and the effects on ARNTL2 and NPAS2 were studied with RT-qPCR and immunofluorescence staining. The role of NF-κB was analyzed using IKK-2 inhibitor IMD-0354. TNF promoted ARNTL2 localization into the nuclei. Similar to *DEC2*, the effects of TNF on *ARNTL2* and *NPAS2* expressions were mediated via NF-κB. Cloned *ARNTL, ARNTL2, NPAS2, DEC1* and *DEC2* were transfected into HEK293. The ARNTL2/NPAS2 dimer was a weaker inducer of *PER3* and *DBP* than ARNTL/NPAS2. ARNTL2 and NPAS2 are regulated by TNF via the same mechanism as DEC2. Compared to their paralogs they have unique effects on other circadian components. Our data suggest that these genes are responsible, at least in fibroblasts, for the accurate adaptation of circadian timekeeping in individual cells during inflammation.

## Introduction

Circadian rhythms have been observed in a variety of organisms ranging from bacteria to humans [1,2]. Every nucleated human cell has a molecular circadian clock that drives a cellular 24-hour cycle and is responsible in keeping up the time in circadian oscillators. In mammals, including humans, the circadian system is organized in a hierarchy of oscillators. The central oscillator is situated in the suprachiasmatic nucleus of the hypothalamus. It controls the peripheral oscillators by neuronal and hormonal mechanisms. External cues such as light and food adjust the circadian rhythm of the central oscillator, which subsequently synchronizes the peripheral ones [2].

The intracellular molecular clock consists of several core genes, which are essential for the generation and regulation of circadian rhythms within individual cells. The primary feedback loop consists of transcription factors *aryl hydrocarbon receptor nuclear translocator like* (*ARNTL*), *circadian locomotor output cycles kaput* (*CLOCK*) [3] and their respective paralogs *aryl hydrocarbon receptor nuclear translocator like 2* (*ARNTL2*) and *neuronal PAS domain protein 2 (NPAS2)* [4]. ARNTL and CLOCK proteins form heterodimers that bind to E-box regulatory elements and drive the expression of clock controlled genes [3]. ARNTL2 and NPAS2 proteins can also form heterodimers with each other and with CLOCK or ARNTL, respectively and induce gene expression through E-box elements [3,5,6,7]. However, their function is less studied compared to ARNTL and CLOCK.

ARNTL/CLOCK heterodimers upregulate the expression of three *period* (*PER1, PER2* and *PER3*) and two *cryptochrome* (*CRY1* and *CRY2*) genes [8,9]. PER/CRY dimers translocate into nucleus and interact with ARNTL and CLOCK heterodimers to inhibit their function. Thus, PER/CRY dimers repress the transcription of clock controlled genes including their own transcription and form a negative feedback loop.

ARNTL/CLOCK also induce another regulatory loop by increasing the expression of *nuclear receptor subfamily 1 group D member* 1 (*NR1D1* or *REV-ERBA*) and *RAR related orphan receptor A* (*RORA*). RORA and NR1D1 compete to bind retinoic acid-related orphan receptor elements (ROR binding elements; RRE’s) at *ARNTL* promoter. RORA and other RORs drive the expression of ARNTL whereas NR1D1 as well as NR1D2 represses this process [10,11,12]. The function of these proteins is necessary for the correct oscillating expression of *ARNTL*.

*Basic helix-loop-helix family member e40* (*BHLHE40* or *DEC1*) and *basic helix-loop-helix family member e41* (*BHLHE41* or *DEC2*) are regulated by ARNTL/CLOCK heterodimer [13,14]. Subsequently, either DEC1 or DEC2 can inhibit the activity of ARNTL/CLOCK by directly binding to E-box elements, and thus both are involved in the fine-tuning of circadian functions such as control of circadian period, clockwork resetting and entrainment in the intracellular molecular clocks [15].

Interestingly, it appears that inflammation and the circadian system are intimately connected. Components of the circadian clock modulate immune cell functions and as such their disturbed expression may lead to immune disorders. On the other hand, inflammatory stimuli affects the function of the clock [16,17,18,19,20,21,22]. We have previously shown disturbed circadian rhythm in rheumatoid arthritis at cellular level. The most disturbed genes throughout the experiments were *ARNTL2* and *NPAS2* [19]. In primary human fibroblasts, TNF stimulus induced the expressions of *ARNTL2* and *NPAS2* but it also caused paradoxical suppression of clock output genes *D-box binding PAR bZIP transcription factor* (*DBP*) and *PER3*. This led us to study the effect of TNF on the expression of *DEC* genes. We showed that TNF increases the expression of *DEC2* possibly explaining the paradoxical downregulation of *DBP* and *PER3* [22]. We hypothesized that DEC2 affects the function of ARNTL2/NPAS2 heterodimer. This was tested by transfecting HEK293 cells with ARNTL2/NPAS2 and *DEC2* and monitoring the effect of the transfections on the expressions of other clock genes.

## Materials and methods

### Subjects

The research plan and the study were approved by the ethical committee of the Helsinki University Hospital (Dnro 165/E6/03). Written informed consent from each patient was obtained to collect sample for research purposes. Guidelines of the Declaration of Helsinki were followed.

### Cell culture

Primary human fibroblast cultures were established and characterized as previously described [22,23]. After establishment, the cells were cultured in RPMI-1640 medium (Lonza Group, Basel, Switzerland) containing 10% fetal bovine serum (FBS; Lonza) 100 IU/ml penicillin and 0.1 mg/ml streptomycin and used in passages 4–5. Stimulation and inhibitor experiments were performed with three different donor fibroblasts.

The synchronization of the molecular clock in cells was performed as described elsewhere [22]. Briefly, tissue samples were minced into small pieces and explants were left overnight in RPMI-1640 medium containing 10% fetal bovine serum with 1000 U/ml penicillin and 1 mg/ml streptomycin solution and on next day changed to basal RPMI with 10% FBS media and 100 U penicillin and 0.1 mg streptomycin.

HEK293 cells were cultured in DMEM medium (Thermo Fisher Scientific, Waltham, USA) containing 10% FBS (Lonza) with 100 IU/ml penicillin, 0.1 mg/ml streptomycin.

### Cell stimulation

For immunofluorescence human primary fibroblasts were seeded on 24-well plates at 4 × 10^4^ cells per well in RPMI-1640 containing antibiotics and 1% FBS for 24 h after which at t = 0 the media was replaced with RPMI-1640 containing antibiotics, 1% FBS and TNF (10 ng/ml; R&D Systems, Minneapolis, USA) or with media containing no added stimulants. After 24 h the cells were washed with PBS and fixed with 4% PFA.

For inhibitor experiment IKK-2 inhibitor IMD-0354 (cat# I3159; Sigma-Aldrich Corporation, St. Louis, USA) was used. 24 h after plating the cells, the media was replaced with RPMI-1640 containing antibiotics, 1% FBS, and IMD-0354 (dissolved in DMSO) in a final concentration of 1 μM or DMSO for 20 minutes. After 20 minute pretreatment (t = 0) TNF (R&D Systems) was added to the wells to a final concentration of 10 ng/ml. After 16 h the wells were washed with PBS and cells were lysed with 350 μl RLT lysis buffer (Qiagen, Hilden, Germany).

### RNA isolation, cDNA synthesis and quantitative real-time PCR

RNA was isolated using RNeasy kit (Qiagen) according to the manufacturer’s instructions. RNA concentrations were measured using NanoDrop ND-1000 instrument (Thermo Fisher Scientific). The cDNA synthesis was performed using 500 ng of total RNA and iScript™cDNA Synthesis Kit (Bio-Rad Laboratories, Hercules, USA) in a 20 μl reaction volume. After cDNA synthesis the cDNA was diluted to 1:5. Quantitative real-time PCR was performed from diluted cDNA in iQ™ SYBR® Green Supermix (Bio-Rad) using gene specific primers (Table 1) in 20 μl reaction volume. The PCR was performed in iQ5 real-time PCR detection system (Bio-Rad). *RPLP0* was used as a housekeeping gene.

**Table 1:** Primers used in quantitative RT-PCR.

Gene	GeneBank Accession	5’ Primer	3’ Primer	Length

*ARNTL2*	NM_020183	GCTAGAGGCTACCAGGCAAAACC	GGTCCACTGGATGTCACTGAAGTC	193
*NPAS2*	NM_002518	CTTCCCTGCCTCCCAACCATC	GGTCCCTGGCTGTTGTGAGTAG	151
*DEC1*	NM_003670.2	TCAGCAGCAGCAGAAAATCATTGC	GTGGGTGACAAGCTGCGAAGAC	187
*DEC2*	NM_030762	TGCTTTACAGAATGGGGAGCGATC	CCCTGGGTGTCCAGCTCTCAAAC	134
*CRY1*	NM_004075	TCTGGCATCAGTACCTTCTAATCC	CTGTGTGTCCTCTTCCTGACTAG	226
*CRY2*	NM_021117	GGTGAAGAACTCAGCAAACGG	ACACACATGCTCGCTCTATCTC	189
*DBP*	NM_001352	CTTAAGCCCCAGCCAATCATGAAG	CCGCCCGCACCGATATCTG	160
*PER1*	NM_002616	CTCCAATCAGGACGCACTTTC	GCTGCCAAAGTATTTGCTTGTG	211
*PER2*	NM_022817	TGTAGGGGCGGACTGCAAAC	TGCTGGTATGACTTGTGTCACTAC	251
*PER3*	NM_016831	TGAAGAATCCATCCCATCCTACTG	TATACTGCTGTCGCTGCTTCC	218
*NR1D1*	NM_021724	CTTGGCTGCCCAGCGTCATAAC	CCAGATCTCCTGCACCGTTCG	274
*RORA*	NM_134262.2	CCAGCCCCGACGTCTTCAAAT	GCCATGAGCGATCTGCTGACA	150
*JUNB*	NM_002229.2	CCACTGGGGTCCAGGGAGCA	GGACTGGGCGCAGGGTAGGA	99
*IL-1β*	NM_000576	TGGCAATGAGGATGACTTGT	GGAAAGAAGGTGCTCAGGTC	237
*RPLP0*	NM_001002	GGCGACCTGGAAGTCCAACT	CCATCAGCACCACAGCCTTC	149

### Immunofluorescence

Human primary fibroblasts were seeded at 1 × 10^5^ cells per well on coverslips placed in 12-well plates containing RPMI-1640 supplemented with antibiotics and 1% FBS. Before stimulations the cells were synchronized as described above. For cellular stimulation the media were replaced with RPMI-1640 containing antibiotics and 1% FBS, without or with 10 ng/ml TNF (R&D Systems). After 24 h cells were washed with PBS and fixed in 4% PFA for 15 min at RT. Fixed cells were permeabilized with 0.1% Triton-X in PBS for 10 min at RT, blocked with 1% BSA-PBS for 1 h at RT, after which slides were incubated with 4 μg/ml rabbit anti-human ARNTL2 IgG (Santa Cruz Biotechnology, Dallas, USA; cat# sc-98300 X) or 4 μg/ml non-immune rabbit IgG at 4°C overnight. Next day slides were incubated in 1:100 dilution of Alexa Fluor 568 labeled goat anti-rabbit IgG secondary antibody (Molecular Probes, Leiden, The Netherlands; cat# ab175471) for 1 h at RT, counterstained in 5 μg/ml DAPI and mounted.

### Plasmids and vectors

*DEC1* (NM_003670.2), *DEC2* (NM_030762.2), *ARNTL* (NM_001178.5), *ARNTL2* (NM_001248004.1) and *NPAS2* (NM_002518.3) cDNA were amplified from human primary fibroblast total cDNA. *ARNTL* and *ARNTL2* were inserted into pDsRed-Monomer-N1 vector (Takara Bio, Kusatsushi, Japan), during insertion DsRed-Monomer was cleaved. *DEC1, DEC2* and *NPAS2* were inserted into pcDNA3.1 V5 hisA vector (Thermo Fisher Scientific). The following primers were used for cDNA amplifications: *DEC1* sense 5’-GCCCCGAAGCTTGCCACCATGGAGCGGATCCCCAGCGCGCA-3’ antisense 5’-ATCCCCGCGGCCGCTTAGTCTTTGGTTTCTAAGTTTAAAGGGGGGA-3’, *DEC2* sense 5’-AACGAAGGATCCGCCACCATGGACGAAGGAATTCCTCATTTGCA-3’ antisense 5’-GGACGCCTCGAGTCAGGGAGCTTCCTTTCCTGGCT-3’, *ARNTL* sense 5’-TCAGATGGATCCGCCACCATGGCAGACCAGAGAATGGACAT-3’ antisense 5’-GCAACAGCGGCCGCTTACAGCGGCCATGGCAAGTCACTA-3’, *ARNTL2* sense 5’-GTGGCTGGATCCGCCACCATGGCGGCGGAAGAGGAGGCT-3’, antisense 5’-CAACAGCGGCCGCCTAGAGGGTCCACTGGATGTCACTGA-3’, *NPAS2* sense 5’-AACTGCAAGCTTGCCACCATGGATGAAGATGAGAAAGACAGA-3’, antisense 5’-AGTGCCCTCGAGTTATCGGGGCGGCTGCTGGAGGCCT-3’.

2.3 kb part of *PER3* promoter (NG_046850.1) was amplified from Human Genomic DNA (Roche Basel, Switzerland; cat# 11691112001) and inserted into pGL3-Enhancer vector (Promega Corporation, Fitchburg, USA). The following primers were used for amplification: sense 5’-ACCTGGTACCCACGCAATAAATGCTTGCTGAACGA-3’ and antisense 5’-CGCGAAGCTTCTCGAGGTCTCCGCGGGGCTCCA-3’.

E-box promoter consisted of three E-boxes within 1.4 kb of upstream of the human *PER1* gene with 6 bp immediate flanking sequences linked together. Following primers were directly annealed sense 5’-CTTAGGCCACGTGACAGTGCGGTCACACGTGGACCCTCAGGTCCACGTGCGCC-CGA-3’ and antisense 5’-AGCTTCGGGCGCACGTGGACCTGAGGGTCCACGTGTGACCGC-ACTGTCACGTGGCCTAAGGTAC-3’ and inserted to pGL3-Enhancer vector (Promega).

For dual-luciferase assay the control vector was pRL-TK (Promega). Vectors were propagated in competent TOP10 *Escherichia Coli* cells (Thermo Scientific). Ultrapure endotoxin-free plasmid DNA was prepared using NucleoBond® Xtra Midi EF (Macherey-Nagel, Düren, Germany) according to the manufacturer’s instructions. Plasmid DNA was diluted in a sterile water. All of the constructs were validated by using nucleotide sequencing (Sequencing Core Facility, Haartman Institute, Helsinki, Finland).

### Transfection

HEK293 cells were seeded on 24-well plates at 4 × 10^4^ cells per well in 0.5 ml DMEM medium and incubated for 24 h before transfection. For transfection, Fugene HD transfection reagent (Promega) was used according to manufacturer’s instructions with total 500 ng DNA and DNA:Fugene HD ratio of 1:3. Amount of *DEC1, DEC2, ARNTL, ARNTL2* and *NPAS2* expression plasmids was 167 ng and if necessary total DNA was adjusted to 500 ng with empty pcDNA3.1 V5 hisA vector. All cell manipulations and assays were carried out 48 hours after transfection.

### Luciferase assay

Transfection of HEK293 cells was carried out as described using 167ng of *DEC1, DEC2, ARNTL, ARNTL2* and *NPAS2* expression plasmids and if necessary total DNA was adjusted to 500 ng with empty pcDNA3.1 V5 hisA vector, 10 ng of reporter plasmid and 1 ng of Renilla luciferase plasmid. Luciferase assay was done using Dual-Luciferase® Reporter Assay System (Promega, cat# E1910) according manufacturer’s instructions 48 h after transfection. Luminescence was measured using Plate CHAMELEON V Multilabel Microplate Reader (Hidex, Turku, Finland).

### Statistical analysis

The means of the IKK-2 inhibitor experiment with two independent samples were tested using student’s t-test. Transfection experiments were analyzed with one-way ANOVA. Significance was tested using Tukey’s post hoc test. Tests were performed with SPSS 21 for Windows (SPSS Inc. Chicago, IL).

## Results

### TNF regulates the core clock components via NF-κB

**Figure 1:**
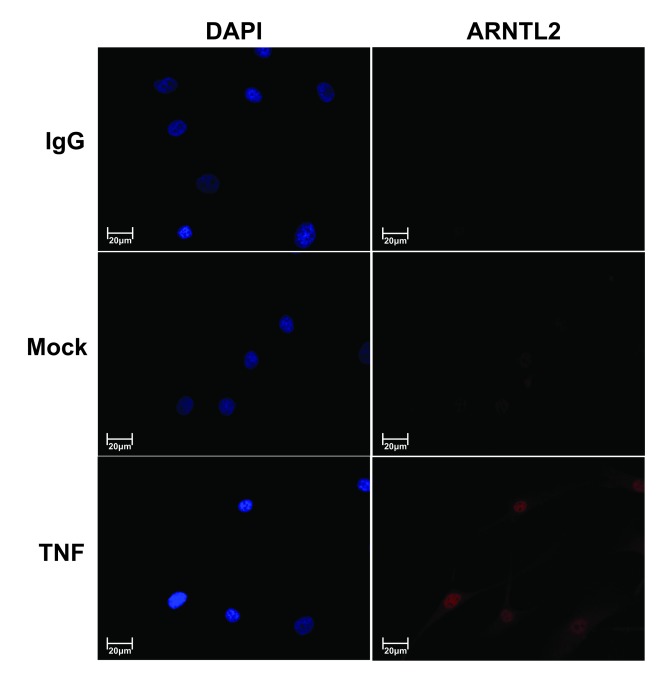
**TNF induces nuclear ARNTL2 expression.** Human primary fibroblasts were stimulated with TNF (10 ng/ml) or PBS for 24 h. ARNTL2 is labeled red and nuclei of the cells are visualized with DAPI (blue).

We have previously shown that TNF induces the expression of *ARNTL2, NPAS2* and *DEC2* and verified that DEC2 is increased at the protein level [19,22]. The increased protein is mainly localized in the nuclei of TNF stimulated cells. To demonstrate that the effect of TNF on the core components is also evident at protein level, human synovial fibroblasts were stimulated with TNF and localization of ARNTL2 protein was studied by immunofluorescence staining (Figure 1). After 24 h of TNF stimulation, ARNTL2 protein was clearly visible and located in the nuclei.

**Figure 2:**
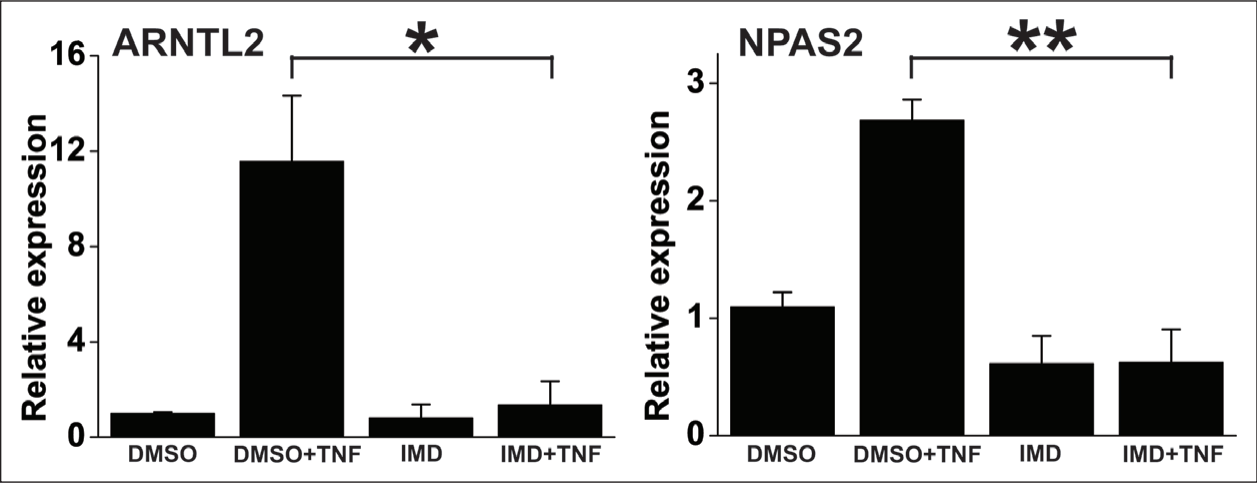
**NF-κB signaling mediates TNF induced ARNTL2 and NPAS2 expression.** NF-κB signaling was blocked in human synovial fibroblasts with 1 μM IMD-0354 (abbreviated IMD) and stimulated with TNF (10 ng/ml) or PBS for 16 h. ARNTL2 and NPAS2 expressions were measured with real time PCR. Values represent means ± SEM of three different experiments performed in duplicate. *p < 0.05, **p < 0.01.

The effect of TNF on *DEC2* expression is mediated by NF-κB pathway [22]. There are clear differences in the kinetics of *ARNTL2, NPAS2* and *DEC2* expression after TNF stimulus [19,22]. Thus, we studied whether NF-κB pathway mediates also the effect of TNF on the expression of *ARNTL2* or *NPAS2*. In the absence of IKK-2 inhibition the expressions of *ARNTL2* and *NPAS2* were increased by 11 and 2 fold, respectively, (Figure 2) after 16 h of TNF stimulation in synchronized human primary fibroblasts. However, inhibition of IKK-2 reduced the TNF induced *ARNTL2* or *NPAS2* expression to basal level demonstrating that TNF mediates its effect via NF-κB signaling.

### ARNTL/NPAS2 and ARNTL2/NPAS2 dimers differently regulate promoter containing canonical E-box element and PER3 promoter

**Figure 3:**
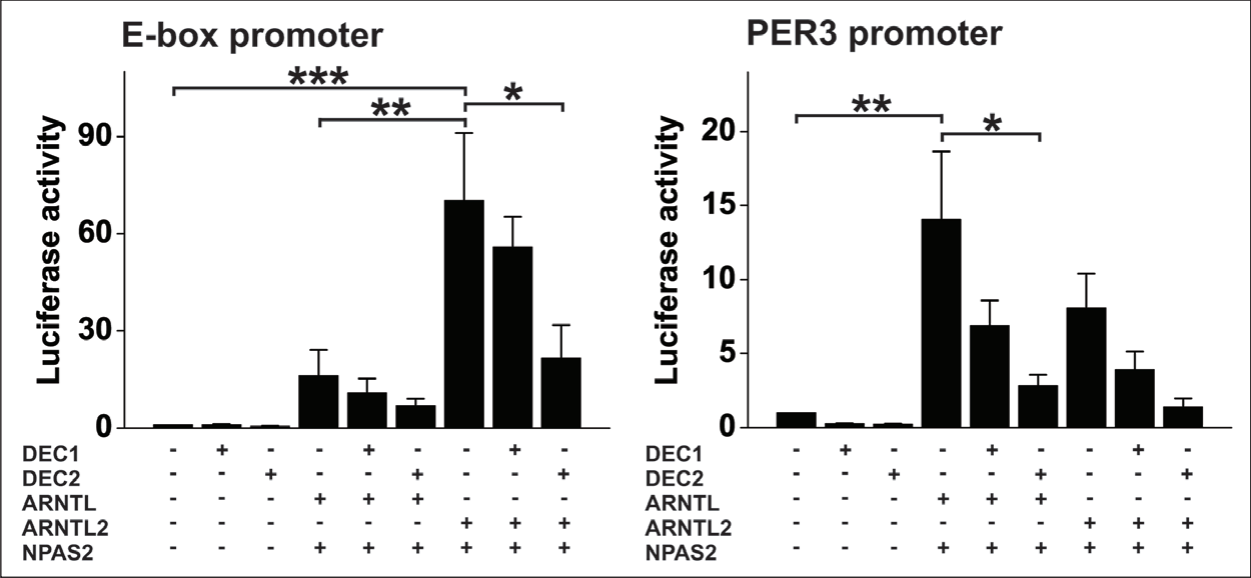
**ARNTL2/NPAS2 dimer is potent activator of E-box element.** HEK293 cells were transfected with empty vector or vector containing DEC1, DEC2, ARNTL, ARNTL2 or NPAS2 in various combinations as shown. E-box and PER3 promoter activities were analyzed using luciferase assay 48 h after the transfection. Values represent means ± SEM of three different experiments performed in triplicate. *p < 0.05, **p < 0.01, ***p < 0.001.

Relatively little is known about the effects of ARNTL2/NPAS2 complex on clock gene expression. Since TNF induces the expression of *ARNTL2* and *NPAS2*, and they are the most disturbed genes in rheumatoid arthritis patients, we investigated how they regulate canonical E-box element compared to more studied ARNTL/NPAS2 complex. Interestingly, ARNTL2/NPAS2 dimer was significantly stronger in inducing pure E-box containing promoter than ARNTL/NPAS2 dimer in HEK 293 cells (Figure 3). ARNTL2/NPAS2 combination increased the luciferase activity over 70 fold versus 16 fold increase by ARNTL/NPAS2 overexpression. However, opposite effect was seen for regulation of *PER3* promoter (Figure 3). We selected to study *PER3* promoter since TNF stimulation reduces the expression of *PER3* more than that of *PER1* or *PER2* [19]. Overexpression of DEC1 and DEC2 suppressed the effect of both ARNTL/NPAS2 and ARNTL2/NPAS2 dimers, however the inhibition by DEC2 was more profound.

### Regulation of gene expression by the combination of ARNTL/NPAS2 or ARNTL2/NPAS2

**Figure 4:**
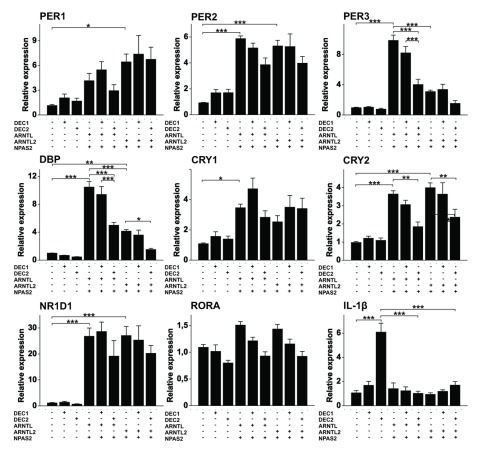
**ARNTL2/NPAS2 dimer is weak inducer of PER3 and DBP in comparison with ARNTL/NPAS2.** HEK293 cells were transfected with empty vector or vector containing DEC1, DEC2, ARNTL, ARNTL2 or NPAS2 in various combinations as shown for 48 h. Expression of the clock components were analyzed by quantitative PCR. Values represent means ± SEM of four different experiments performed in triplicate. *p < 0.05, **p < 0.01, ***p < 0.001.

**Figure 5:**
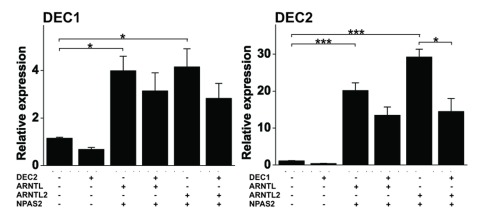
**DEC2 is not able to inhibit DEC1.** HEK293 cells were transfected with empty vector or vector containing DEC1, DEC2, ARNTL, ARNTL2 or NPAS2 in various combinations as shown 48 h. Expression of the clock components were analyzed by quantitative PCR. Values represent means ± SEM of four different experiments performed in triplicate. *p < 0.05, ***p < 0.001.

Based on previous experiment ARNTL2/NPAS2 dimer is capable of inducing gene expression through E-box elements but the effect is evidently dependent on other elements on the promoter as ARNTL2/NPAS2 dimer was weaker activator of *PER3* promoter although it contains two canonical E-box elements. Thus, we wanted to compare the effect of ARNTL/NPAS2 and ARNTL2/NPAS2 on the expressions of *PER, CRY* and *DEC* genes (Figure 4) by transfecting plasmids containing these elements into HEK293 cells. Both *PER1* and *PER2* as well as *CRY1* and *CRY2* were equally responsive to either combinations but the expression of *PER3* was induced more with the combination of ARNTL/NPAS2 when compared to that of ARNTL2/NPAS2 (p < 0.001). This was also true for clock output gene *DBP* (p < 0.001). The results on *PER3* are consistent with the luciferase assay, in which the promoter activity of *PER3* gene was induced more with the combination of ARNTL/NPAS2 in comparison to that of ARNTL2/NPAS2. Both dimers increased the expression of *DEC1* and *DEC2* equally (Figure 5) although there was a trend of ARNTL2/NPAS2 being more potent inducer of *DEC2* expression than ARNTL/NPAS2 (p = 0.058. The most up-regulated gene of the studied genes was *NR1D1* (*REV-ERBA*) (Figure 4) with nearly 30 fold induction. Both ARNTL/NPAS2 and ARNTL2/NPAS2 heterodimers were equally effective in inducing *NR1D1* expression. The expression of *RORA* was not affected by either combination (Figure 4).

### Both DEC1 and DEC2 inhibit the ARNTL/NPAS2 and ARNTL2/NPAS2 complexes

To understand the effect of DEC1 or DEC2 on ARNTL/NPAS2 or ARNTL2/NPAS2 induced gene expression, different combinations of these components were transfected into HEK293 cells. DEC2 repressed the effect of ARNTL/NPAS2 on the expression of *PER2, PER3, DBP*, and *CRY2* (Figure 4). In contrast DEC1 was not able to inhibit the effect of ARNTL/NPAS2 on any of these genes. Similarly DEC2 also inhibited the ARNTL2/NPAS2 induced expression of *PER3, DBP* and *CRY2* whereas DEC1 did not (Figure 4). We have shown previously that DEC2 induces the expression of IL-1β [22]. Interestingly, overexpression of either ARNTL/NPAS2 or ARNTL2/NPAS2 completely neutralized this effect (Figure 4).

### DEC1 inhibits DEC2 expression

DEC1 inhibits the expression of DEC2 through binding to the E-box in the proximal promoter of the gene [24]. We confirm that DEC1 inhibits DEC2 expression but also demonstrate that DEC2 is not able to inhibit DEC1 expression. This is also true when DEC1 or DEC2 expression is induced by ARNTL/NPAS2 or ARNTL2/NPAS2 (Figure 5). It appears that DEC1 is more powerful in repressing DEC2 when compared to the effect of DEC2 on DEC1 in the presence of ARNTL/NPAS2 or ARNTL2/NPAS2. It was impossible to analyze the effects of DECs on their own expressions because endogenous expressions of the genes were masked by the overexpression due to the gene transfections.

## Discussion

Proinflammatory cytokine TNF increases the expression of *ARNTL2* and *NPAS2* in primary human fibroblasts [19]. Of the clock genes, the expression of *ARNTL2* and *NPAS2* is the most perturbed in RA [19], and yet the function of these genes is the least studied among the core clock genes.

In this study, we first confirmed that inflammation increases the nuclear protein levels of ARNTL2. The effect of TNF on *ARNTL2* and *NPAS2* expression was NF-κB dependent. The effect is analogous to that observed on *DEC2* expression. It appears that the same mechanism controls the expression of *DEC2* as well as the expression of *ARNTL2* and *NPAS2*, despite the different kinetics of *ARNTL2, NPAS2* and *DEC2* expression following TNF stimulus [19,22].

Either ARNTL2 or NPAS2 can replace their paralogs in the dimers that bind to E-box elements both *in vitro* and *in vivo* [4,6,7]. However, the function of heterodimer composed of ARNTL2 and NPAS2 is the least investigated in human cells. Our results show that ARNTL2/NPAS2 heterodimer induces robust gene expression through E-boxes. This effect was even stronger than that of ARNTL/NPAS2 when pure E-box elements of the human *PER1* gene were used. However, when 2.3 kb part of the *PER3* promoter was tested the effect on promoter activation was the opposite. The cloned PER3 promoter contains 2 canonical E-boxes in addition to total of 6 non-canonical E-box (CAGGTG, CACGCG, CTCGAG and CACCTG). Most likely ARNTL/NPAS2 and ARNTL2/NPAS2 complexes bind differently to canonical and non-canonical E-boxes. This hypothesis is supported by analysis of the expression of different clock genes after transfecting either ARNTL or ARNTL2 with NPAS2 into cells. We were able to replicate the findings of the luciferase assay. ARNTL2/NPAS2 heterodimers induced *PER1* expression, which was mildly higher than expression induced by ARNTL/NPAS2, whereas the induction of *PER3* mRNA expression was significantly weaker by ARNTL2/NPAS2 when compared to ARNTL/NPAS2. The same held true for *DBP* and to a lesser extent for *CRY1*. In contrast, the ARNTL2/NPAS2 induced expression of *DEC2* was slightly higher than that of ARNTL/NPAS2 although not statistically significant (p = 0.058). The two heterodimers had similar effect on *DEC1, PER2, CRY2, NR1D1*, and *RORA*. Preferential promoter binding may explain our finding as CRYs have a similar inhibitory effect on ARNTL/CLOCK and ARNTL2/CLOCK dimers [25] and ARNTL2/NPAS2 dimer [26] but different heterodimers activate *mPer1* promoter with different efficacy [26]. Our results demonstrate that despite being able to fully compensate each other in the heterodimers, ARNTL and ARNTL2 have different capabilities in driving the gene expression of different clock components depending on the promoter of the gene.

Perplexingly TNF stimulus decreases the expression of *PER3* and *DBP* [19] although their promoter regions are rich in E-box elements and ARNTL2/NPAS2 dimer is capable of driving gene expression through these elements and both the expression of *ARNTL2* and *NPAS2* and the amount of at least ARNTL2 is increased in the nuclei of TNF stimulated cells. The relatively weak effect of ARNTL2/NPAS2 complex in inducing the expression of *PER3* and *DBP* may at least partly explain these findings. However, TNF stimulus does not affect the expressions of *ARNTL* or *CLOCK* (data not shown) and upregulates that of *ARNTL2* and *NPAS2*. Thus, the finding still appears paradoxical. Since TNF also induces the expression of *DEC2*, we hypothesized that this induction of *DEC2* expression leading to nuclear DEC2 accumulation may inhibit the ARNTL2/NPAS2 complex. Indeed, both DEC1 and DEC2 efficiently decreased ARNTL2/NPAS2 driven E-box and *PER3* promoter activities. The observed activity of *PER3* promoter dropped even below control in the presence of DEC2 despite ARNTL2/NPAS2 complex. Although DEC1 was able to inhibit both ARNTL/NPAS2 as well as ARNTL2/NPAS2 complexes the effect was not as apparent as that of DEC2. This is in line with earlier findings with mouse Dec2 being a stronger inhibitor of mouse Arntl/Clock and Arntl2/Clock induced promoter activation than Dec1 [14,27].

We further studied how the presence of DEC1 and DEC2 affects ARNTL/NPAS2 mediated gene expression. DEC2 was more potent in inhibiting ARNTL/NPAS2 induced *PER3, CRY2* and *DBP* expression compared to DEC1. No effect of DEC2 was observed on the expression of *PER1, CRY1* or *NR1D1*. Almost similar effects of DEC1 or DEC2 were observed also on gene expression mediated by ARNTL2/NPAS2 complex with DEC2 being able to significantly inhibit the expression of *PER3, CRY2* and *DBP* induced by ARNTL2/NPAS2.

Taken together these findings demonstrate that DEC2 effectively inhibits E-box driven gene expression. Particularly, the upregulation of *PER3* and *DBP* by either ARNTL/NPAS2 or ARNTL2/NPAS2 complexes are efficiently blocked by DEC2. These data explain at least partly the paradoxical observation we have made previously. On the other hand, this study also suggests that DEC1 is a weaker inhibitor of ARNTL/NPAS2 or ARNTL2/NPAS2 than DEC2.

The best known function of DEC2 is competition with ARNTL/CLOCK to bind E-box elements and therefore inhibit ARNTL/CLOCK. DEC2 has also been shown to have transactivating abilities. In mice, Dec2 binds the promoters of *Gata3* and *Junb* to sequences differing from E-box element [17] to drive their expression. We have demonstrated that DEC2 is capable of driving *IL-1β* expression in primary human fibroblasts with normal genome. Co-transfection of either ARNTL/NPAS2 or ARNTL2/NPAS2 with DEC2 completely abolished this effect on *IL-1β*. Junb deficient bone marrow–derived macrophages have decreased IL-1β production when being LPS stimulated [28]. This suggests that ARNTL/NPAS2 or ARNTL2/NPAS2 could block the effect of DEC2 on *IL-1β* expression through inhibition of DEC2 induced *JUNB* expression. We were not able to confirm that either DEC1 or DEC2 could induce *JUNB* expression (data not shown), thus suggesting that the effect of ARNTL/NPAS2 or ARNTL2/NPAS2 on DEC2 induced *IL-1β* expression may not be JUNB dependent.

These findings indicate redundant functions of core clock genes but also highlight the versatile fine tuning of circadian timekeeping machinery. ARNTL2/NPAS2 regulates differently the expression of *PER3, DBP* and *DEC2* when compared to ARNTL/NPAS2, and DEC1 is not able to substitute DEC2 in controlling *PER3* or *DBP* expression. Strikingly, *ARNTL2* and *NPAS2* were the core clock genes that were found to be disturbed in rheumatoid arthritis and they accompanied *DEC2* by also reacting to TNF with increased expression. *Arntl2* is linked to type 1 diabetes and functions in T-cells [29], *Dec2* is essential for T-cell development [17], and low *PER3* expression is associated to several different cancers and poor prognosis [30,31,32,33,34,35]. Our data indicate that ARNTL2 containing dimers are weaker inducers of *PER3* expression than ARNTL containing dimers and thus higher ARNTL2 expression, as in response to TNF, could lead to decreased PER3 expression through competition for E-box binding [25]. Induction of *DEC2* by TNF could function to further decrease *PER3* expression. It remains to be investigated if low *PER3* or *DBP* have any role during inflammation or tumorigenesis, but it appears important that ARNTL2, NPAS2 and DEC2 work in concert during inflammation to regulate the proper expression of clock controlled genes *PER3* and *DBP*.
